# An investigation of the utility of QuEChERS for extracting acid, base, neutral and amphiphilic species from example environmental and clinical matrices

**DOI:** 10.1002/ansa.202000018

**Published:** 2020-06-05

**Authors:** Rachel Townsend, Geertje van Keulen, Claire Desbrow, Amy Ruth Godfrey

**Affiliations:** ^1^ Swansea University Medical School Swansea UK; ^2^ Biotage GB Limited Hengoed UK

**Keywords:** clinical analysis, environmental solids, liquid chromatography‐mass spectrometry, medicinal compounds, QuEChERS

## Abstract

Accurate measurement of the composition of complex samples is key for the safety and efficacy of a range of products used in daily life, with sample preparation a critical step in this workflow. QuEChERS is one such method, however published protocols do not explicitly address acidic, basic, neutral, and amphiphilic species in a single protocol and often use extra steps or an alternative preparation to recover the breadth of chemical types. Our work addresses this need by investigating the use of QuEChERS for monitoring this wide range of chemistries within environmental solids and blood plasma, using a protocol that can accommodate both milliliter and microliter sample volumes. While published methods can require significant resource and time, our approach offers a reduction in preparation time (for environmental samples), with the “micro‐QuEChERS” protocol offering a further reduction in cost. The analytical performance of these methods were assessed using reversed‐phase LC‐MS and showed good accuracy, precision, and sensitivity for the expected concentrations in the tested applications. Target analytes of variable lipophilicity/acidity were extracted and isolated from soil, with largely repeatable matrix effects < 15%RSD and recoveries of 39‐100%. An initial “proof‐of‐concept” investigation using the “micro‐QuEChERS” protocol showed reduced matrix enhancement (median value of 90%ME) for soil, and improved matrix effects and recovery (>65%) for blood plasma. This novel sample preparation method can therefore offer an improved approach with wider applicability providing “cleaner” extracts than other methods used for high‐throughput clinical analysis.

**Abbreviations**
BAC‐C12: benzyldimethyldodecylammonium chloride
BAC‐C14: benzyldimethyltetradecylammonium chloride
BAC‐C14‐d_7_
: benzyldimethyltetradecylammonium chloride‐d_7_
dSPE: dispersive solid‐phase extraction
HDTMA: hexadecyltrimethylammonium chloride
MgSO_4_
: magnesium sulphate
NaOAc: sodium acetate
PSA: primary secondary amine
QAC: quaternary ammonium compound
QuEChERS: Quick, Easy, Cheap, Effective, Rugged and Safe

## 1 INTRODUCTION

Advances in the analytical workflow have largely concerned instrumentation accuracy and sensitivity^1^; however, there remains a need to develop sample preparation technology to meet the demands of “blue‐sky” research and regulated industry.^2^ Ideally sample preparation strategies will repeatedly provide extracts containing high amounts of the target analyte(s) for measurement without interference, while using low amounts of resource and time to do so.^1^ However, a compromise is often encountered during development with only some of these desirable characteristics achieved, with these depending on whether a targeted preparation or a “screen” of the sample for unknown materials (i.e. biomarker studies) is required.^1^ Example complex mixtures include environmental and clinical samples and these have traditionally used multistep preparations with a range of procedures and equipment, resulting in time and resource consuming methods.^1^

With greater focus on links between environmental and public health, and increased regulation for pollution and waste management, the selection of environmental matrices, target chemistries and their (trace) amounts for analysis^3^, ^4^, ^5^ make the analytical challenge more difficult to address without appropriate sample preparation solutions. For example, the introduction of the Water Framework Directive^3^ and Circular Economy legislation (waste and landfill^6^) has increased the types of matrices requiring molecular (pollutant) characterization to include organisms, wastewater, sludges, and receiving sediments.^3^ These regulations are evidence‐driven with substances of concern and their environmental limits^4^, ^5^ continually evolving to protect environmental and public health. With eight pharmaceuticals considered as pollutants on a legislative “watch list”^5^, ^7^ the scope of monitoring programs has expanded to include other medicinal analytes within waste materials.^8^ These may cover a range of classes with high usage (and perceived disposal) rates (e.g. diphenhydramine, carbamazepine, erythromycin, etc) and lipophilicity (log *K*_ow_, log *P*, or *K*_d_), with the latter enabling sorption to sludge and/or bioaccumulation within soil and biota, respectively.^9^, ^10^ Given the significant re‐use of treated sewage sludge as agricultural fertilizer (80%)^11^ and the proposed effects on agricultural soil (e.g. antimicrobial resistance^12^), it is essential that pollutants are accurately measured within these samples.

Recognized methods for analyzing environmental matrices such as soil and wastewater are generally laborious multistep procedures using a range of techniques and equipment, rendering them unsuitable for high‐throughput analysis.^9^, ^10^ Environmental solids are particularly challenging due to the myriad of analytes present and the sorption of trace material to more abundant species (e.g. humic acids); sample preparation therefore requires additional stages to displace the trace analyte resulting in some extractions taking hours per sample.^9^, ^10^ When tested in‐house, standard methods have also exhibited poor repeatability for pharmaceuticals (data not shown) further highlighting the need for a simple, rapid, and reliable sample preparation with minimal matrix effects on the measured signal and high recovery. Similar to environmental samples, clinical matrices are complex with target molecules (such as pharmaceuticals) often present in trace amounts.^1^, ^2^ Again, a compromise is often encountered between a more targeted multistep preparation to ensure highly accurate and sensitive measurements, or a quick method to remove an impurity class (e.g. protein precipitation) that relies on instrumentation sensitivity, often resulting in increased matrix interference and instrument down‐time due to fouling.^1^, ^13^ Therefore, for qualitative screening of clinical matrices (e.g. biomarker studies) where an unbiased but quick approach is required (given the substances of interest are unknown), there remains a need for a low cost, quick preparation method capable of extracting a range of analytes at trace concentrations without fouling downstream instrumentation.

The QuEChERS sample preparation method is a two‐step process originally developed for the extraction of acidic and basic pesticides from foodstuffs,^14^ involving a liquid extraction (typically using acetonitrile) and a dispersive solid‐phase extraction (dSPE) using primary‐secondary amine (PSA), C18, and/or graphitized carbon black (GCB) sorbents to target the removal of abundant interferences (e.g. humic acids, lipids, etc). This less biased approach provides the distinct advantage of method flexibility for screening environmental and clinical samples and is evidenced by recent work concerning specific analyte classes (e.g. pharmaceuticals^15^, ^16^, ^17^, ^18^, ^19^, ^20^, ^21^, ^22^ and surfactants^23^, ^24^, ^25^, ^26^), and environmental^15^, ^16^, ^17^, ^21^, ^22^, ^24^, ^27^, ^28^, ^29^, ^30^ and clinical matrices.^19^, ^20^, ^26^, ^31^, ^32^, ^33^, ^34^ However, these studies have been varied in their approach and have not explicitly addressed the breadth of analyte classes as a single method in samples such soil and blood plasma without using additional steps to the protocol or alternative preparations to recover the analytes. Therefore, there remains a need to understand the chemical breadth of QuEChERS for extracting acidic, basic, neutral, and amphiphilic analytes from these environmental and clinical matrices as a single preparative protocol. Pilot work has also shown potential to reduce the scale of QuEChERS to minimize resource (e.g. two pharmaceuticals in biota,^17^ bisphenol A in urine,^33^ and pesticides in tissues^32^); however, the capability of (micro‐)QuEChERS in extracting a range of acidic, basic, neutral, and amphiphilic medicinal analytes from blood plasma again remains unknown.

Given its successful application to similar complex samples and the extraction of selected pharmaceuticals and pesticides, we believed QuEChERS would provide an alternative single protocol extraction for the range of anticipated chemistries (acidic, neutral, basic, and amphiphilic) in soil and plasma, as a quicker and cheaper approach, with less matrix interference versus traditional methods described above. In readiness for the eventual application, the sample preparation was developed as part of a quantitative analytical method using an internal standard (IS) approach. For smaller‐scale extractions (potential high‐throughput formats and automation), the optimized protocol was also investigated as a “micro‐QuEChERS” approach for implementation at lower cost.

## 2 METHODS AND MATERIALS

### 2.1 Chemicals and materials

To represent acidic, basic, and neutral pharmaceuticals diclofenac sodium, loratadine, acetaminophen, carbamazepine, citalopram hydrobromide, propranolol hydrochloride, fluoxetine hydrochloride, diphenhydramine hydrochloride, and erythromycin were purchased from Sigma–Aldrich (Poole, UK) and assigned according to their predicted state at pH 7 (see Supporting Information). For the amphiphilic (surfactant) class, quaternary ammonium compounds (QACs) were purchased from Sigma–Aldrich (Poole, UK) and included benzyldimethyldodecylammonium chloride (BAC‐C12), benzyldimethyltetradecylammonium chloride (BAC‐C14), and an aliphatic surfactant hexadecyltrimethylammonium chloride (HDTMA). While as ISs acetaminophen‐(*methyl*‐d_3_) and 10,11‐dihydrocarbamazpine, pronethalol hydrochloride, and talopram hydrochloride were purchased from Tocris (Abingdon, England) and benzyldimethyltetradecylammonium chloride‐d_7_ (BAC‐C14‐d_7_) from Toronto Research Chemicals (Ontario, Canada). For sample preparation, modified QuEChERS extraction kits were obtained from Biotage (Uppsala, Sweden) with formic acid, acetonitrile, and water (HPLC‐grade) from Fisher Scientific (Loughborough, UK). As complex matrices for proof‐of‐concept testing, garden soil was collected from an undisclosed location in West Wales and blood plasma sourced from Seralab (West Sussex, UK).

### 2.2 Instrumentation

Chromatographic separation was carried out using a Thermo Scientific (Hemel Hempstead, UK) liquid chromatography (LC) system consisting of a Micro AS autosampler and MSPump Plus, with a reversed phase Waters (Elstree, UK) Xselect HSS T3 LC column (1 × 100 mm, 3.5 μm) and a Phenomenex KrudKatcher Ultra online filter (Macclesfield, UK). The LC system was controlled using Xcalibur 2.0.7 software (Thermo Scientific) with detection performed using a dual mass spectrometry approach to accommodate the range of scan speeds and sensitivities anticipated for the differing analyte concentrations within the sample types (established from in‐house screening). For the more abundant amphiphilic analytes, a Thermo Finnigan LCQ ion trap mass spectrometer (Hemel Hempstead, UK) was operated with an ESI source in positive ion mode using Xcalibur 2.0.7 software, while, for the broader suite of pharmaceuticals, a faster scanning Waters Micromass ZQ4000 single quadrupole (Manchester, UK) mass spectrometer, again operating with an ESI source in positive ion mode, was used to capture sufficient data for quantitation using MassLynx 4.0 software. Data processing was undertaken with relevant instrumentation software and Microsoft Excel 2010.

### 2.3 Stock and working solutions

Where possible stock solutions (1 mg/mL) were prepared in water to minimize degradation during storage while erythromycin, loratadine, carbamazepine, 10,11‐dihydrocarbamazepine and QACs were prepared in 100% acetonitrile due to limited water solubility. Calibration standards were made from the stock solutions as a pharmaceutical and QAC mixture in 50:50 acetonitrile/water (1‐400 ng/mL for each pharmaceutical (apart from acetaminophen (5‐400 ng/mL) and 2‐80 ng/mL for QACs) with a relevant internal standard (100 ng/mL for pharmaceuticals and 20 ng/mL for QACs). Quality control (QC) samples to test the quantitative performance of the method were similarly prepared at four concentrations within the calibration range; 15, 25, 100, and 350 ng/mL for pharmaceuticals; and 8, 20, 60, and 80 ng/mL for QACs. A “double” blank (S_B_) containing just solvent and an IS blank (S_0_) were also included to confirm method selectivity by detecting sample carryover and analyte contamination, respectively.

### 2.4 Sample preparation conditions

A novel QuEChERS method was developed using 4 g magnesium sulphate (MgSO_4_) and 1.5 g of sodium acetate (NaOAc) as the initial extraction step, followed by dSPE using 150 mg PSA and 900 mg MgSO_4_. The extraction performance was assessed by determining matrix effects (%ME), recovery (%REC), and process efficiency (%PE), comparing samples whereby analyte is spiked before extraction (SBE), after extraction (SAE) and a non‐extracted standard of equivalent concentration^35^ (see Supporting Information for details). Triplicate samples containing a mixture of pharmaceutical and QAC analytes at a concentration of 100 ng/mL and 60 ng/mL (and relevant IS) were extracted; 10 mL of acetonitrile and the contents of the initial extraction tube were added to each sample, shaken for 1 min, centrifuged for 5 min, with the supernatant added to the dSPE material, and vortexed for 1 min and centrifuged for a further 5 min. The post‐extraction supernatant was transferred to a fresh vial, evaporated to dryness under nitrogen, and reconstituted in 50:50 water/acetonitrile. While for “micro‐QuEChERS”, a simplified approach of reducing all sample volumes, reagents, and extraction media by a scaling factor of 1/8 was used.

### 2.5 Liquid chromatography‐mass spectrometry analysis

Samples were kept at 4°C prior to injecting 5 μL on‐column and separated using a gradient elution program of 0.1% formic acid in water (A) and acetonitrile (B) at a flow of 50 μL/min (95% A:5% B at 0‐2 min, with %B increasing linearly over 28 min) based on prior studies^15^, ^36^ and an in‐house general screening method. Prior to method development the mass spectrometers were optimized for acquiring the target precursor and fragment ions using a combination of full mass scan and selected reaction/ion monitoring modes to facilitate qualitative and quantitative analyses (see Supporting Information for details). The integrated peak area from the relevant ion chromatogram was used to evaluate the sample preparation and generate calibration graphs over selected concentration ranges (estimated from in‐house sample screening) to confirm the amounts recovered from the extraction (see Supporting Information).

## 3 RESULTS AND DISCUSSION

### 3.1 Analytical method characterization

#### 3.1.1 Analytical selectivity and chromatographic repeatability

Each target analyte and internal standard was identified by the anticipated protonated molecule, isotope pattern, and chromatographic retention time; only diclofenac and talopram (IS) did not exhibit mass selectivity (*m/z* 296) however, these had notable differences in isotope pattern (chlorine) and baseline chromatographic separation indicative of their different hydrophobicity on column. Given this dependence on chromatographic selectivity, retention time repeatability was determined using sequential injections of a standard mixture over 2 days and by calculating the relative standard deviation (%RSD) and variance (using a two‐tailed *F*‐test), respectively. The data showed repeatable chromatography for all analytes (with no detectable carryover) on both days (RSDs < 5%) and no significant difference determined between the 2 days; largely chromatographic stability improved for acidic/neutral/basic analytes with prolonged use (lower %RSDs for day 2, see Supporting Information) indicating a further settling of chromatographic conditions with time. For amphiphilic analytes stable chromatography was also observed, with typical repeatability of <0.23%RSD and no significant difference in chromatography over separate days of analysis. To understand the error associated with sample injection for method quantitation, injection repeatability was also determined using the chromatographic peak area for each analyte. Again, good repeatability was observed (<12%RSD) with stable chromatographic and ionization conditions for assessing quantitative performance (see Supporting Information).

#### 3.1.2 Calibration statistics, limit of detection (LOD), precision, and accuracy

To establish if adjustment of the regression function was required to accommodate any heteroscedasticity within the calibration data, the percentage relative error (%RErr) of the calculated and theoretical concentrations for selected weighting factors was determined, and the simplest weighting factor of lowest %RErr^37^ with *R* ^2^ ≥ 0.98 chosen as the most suitable regression function. As expected for the selected instrumentation, 1/*x* proved the most suitable for analyte quantitation (see Supporting Information) with good linearity observed over the measured dynamic range(s) for most analytes following adjustment. For the LOD, values were initially calculated using statistical and empirical methods, however, the former method assumes data are homoscedastic^38^ and so was omitted for further work. Pleasingly, empirical LODs showed good sensitivity at <1 ng/mL apart from acetaminophen and HDTMA (5.81 and 1.88 ng/mL, respectively) however, all were considered suitable for the anticipated concentration range within relevant samples (in‐house data). For quantitation, the method precision and accuracy were characterized using replicate QCs at four concentrations within the dynamic range (see Supporting Information for more detail). Despite the higher precision values observed for the amphiphilic species (indicative for ion trap data), the method remained reliable for quantifying within the concentration range with largely good precision and accuracy determined for each analyte (≤20.5%RSD and mean accuracy < 16.0%) at all QC concentrations.

### 3.2 QuEChERS sample preparation method evaluation

#### 3.2.1 Method optimization

**FIGURE 1:**
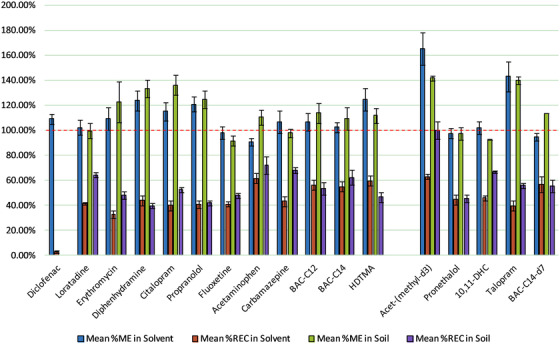
Comparison between the mean percentage matrix effects and recovery (with standard error bars) of acidic, neutral, basic and amphiphilic analytes calculated for the spiked solvent and spiked soil samples following QuEChERS extraction (n = 3). The dashed line indicated the zero matrix interference of the signal (i.e. 100% matrix effect)

Initial feasibility and optimization of the QuEChERS extraction for acidic, neutral, basic, and amphiphilic analytes were carried out using a simplified water matrix and evaluated according to the duration of extraction (as an assessment of cost), matrix effects (%ME), analyte recovery (%REC), and overall process efficiency (%PE), where the method would ideally exhibit reliably low matrix interference and high recovery (both with RSD < 20% relevant for the tested applications) of the measured analyte for high process efficiency. Given the minimal equipment required for QuEChERS, multiple samples were rapidly extracted (facilitating replicate analyses), enabling a reduction in analyst time (from hours to ∼20 min per sample) and a saving of extraction costs of >60% (versus recommended solid phase extraction cartridges^10^) for environmental solids. From an initial study with the commercially available buffered EN extraction method, the presence of sodium chloride (counter ion) resulted in undesirable peak broadening and poor recovery for the majority of acidic, neutral, and basic species, and therefore, alternative reagent mixtures were investigated. Of these, 4 g of MgSO_4_ with 1.5 g NaOAc (a mixture of the EN and AOAC extraction) followed by PSA dSPE (to remove acidic interferences anticipated in the sample matrices) with MgSO_4_ proved promising, showing repeatable sample preparation for most analytes selected (%RSD ≤20%) and minimal matrix effects (median of 106%ME) as shown in Figure 1. The exceptions to this were acetaminophen‐(*methyl*‐d_3_), talopram, diphenhydramine, propranolol, and HDTMA, where matrix enhancement was observed; however, given the good repeatability (%RSDs < 14%) for all analytes this signal enhancement may be accounted for, ensuring a more accurate analyte recovery and process efficiency. This novel QuEChERS blend also showed repeatable recovery (%RSD < 20%) for the majority of analytes apart from diclofenac; while the result for diclofenac is unsurprising (i.e. likely retention on the cationic dSPE at the sample pH (<8)), the remaining data indicates a high level of robustness for alternative chemistries. Pleasingly, QuEChERS also offers potential to mitigate this loss, with flexibility to include alternative dSPE material (such as anion exchange given low analyte recoveries observed when using C18 in‐house) for the parallel clean‐up of the initial supernatant. Interestingly, when considering the remaining analytes all classes had similar mean recoveries (∼40%) with slightly higher values observed for amphiphilic species (∼55%); this again, supports the use of QuEChERS as a preparative method for analyzing different chemistries as a screen, with reasonable process efficiency and little optimization.

#### 3.2.2 Application of the novel QuEChERS to complex samples: Environmental solids

The novel QuEChERS extraction was tested using locally sourced garden soil as an example control matrix for contaminated agricultural soil or wastewater sludge cake. These sample matrices typically contain large amounts of non‐volatile substances such as inorganic salts and macromolecular organic structures that include fulvic and humic acids. These latter species can behave as acidic substances (negatively charged), contributing to signal suppression of the target analytes,^39^ and be particularly problematic for those with nonpolar character given they can elute at similar chromatographic conditions. Admittedly, there is significant body of work investigating QuEChERS for the extraction of pesticides from soils however, those concerning a broader range of target analytes are typically partial QuEChERS protocols (i.e. initial extraction) with relevant analytes prone to significant suppression, even when combined with further preparative steps such as a heptane solvent extraction and cartridge SPE^24^ or even dual cartridge SPE.^22^ Recent studies involving a complete QuEChERS protocol have been undertaken more successfully for veterinary drugs and selected pharmaceuticals in soil; however, these required an additional filtration step and dSPE materials, and signal suppression, while albeit less, remained.^29^, ^30^ Given this, there continued to be a case to investigate the potential improved performance of our novel QuEChERS blend to extract the breadth of chemistries with less suppression and increased sample preparation resource (and time) of a dual SPE protocol.

To test this approach, control soil was fortified with analyte and IS mixtures (apart from diclofenac due to poor recovery in solvent) to determine the matrix effects and recovery. As expected, there was greater evidence of matrix effects for each analyte but, pleasingly most measurements showed little suppression (largely enhancement) with a median value of 113%ME (see Figure 1), supporting our initial hypothesis and dSPE selection for acidic interferences, and improved repeatability with RSDs < 15%. Unfortunately, there is limited published repeatability data for allied work^30^ and so a “true” comparison of performance with similar methods is difficult to undertake; however, given the consistency of performance is necessary for method robustness and “normalizing” the recovery for matrix effects, this precision is highly desirable as it enables the determination of quantified amounts without suppression (or enhancement) adversely influencing the data. Interestingly, there continues to be no apparent bias with the extraction of chemical class using this protocol further supporting the application of QuEChERS as a screening method for samples where the target extraction analyte is undecided or unknown. For the different analytes, recovery was largely repeatable (< 18%RSD) and of comparable, if not, improved performance when extracted from soil. For analytes tested with the standard EPA method within biosolids,^10^ this new protocol also showed improved repeatability of analyte recovery for carbamazepine, diphenhydramine, and fluoxetine, although this was at a compromise of a lower recovery (39.8‐100.1%). However, lower recoveries have been reported for some of the tested analytes in other work using the EPA method, with values, pleasingly, more comparable to this novel QuEChERS protocol.^40^ When compared to established QuEChERS‐based methods that involving additional clean‐up steps,^22^, ^30^ better or comparable recoveries were also observed for most analytes common to these studies, highlighting the potential gain of this method. Admittedly, higher recoveries have been observed for carbamazepine,^22^ citalopram, and fluoxetine^30^; however, these protocols were designed for a more targeted class of analytes, and presented limited repeatability data, making it difficult to fully benchmark to our method.

In summary, this new QuEChERS method has shown potential for extracting the majority of analytes tested with desirable characteristics such as extraction repeatability, minimal inherent matrix effects, reasonable analyte recovery (and process efficiency), and a short extraction time. For use as an environmental protocol, it offers a reduction in analyst time from hours to ∼20 min and extraction costs by >60% (of solely extraction cartridges) per sample, key to the future adoption of the method.

#### 3.2.3 Scalability of the novel QuEChERS method: “micro‐QuEChERS”

**FIGURE 2:**
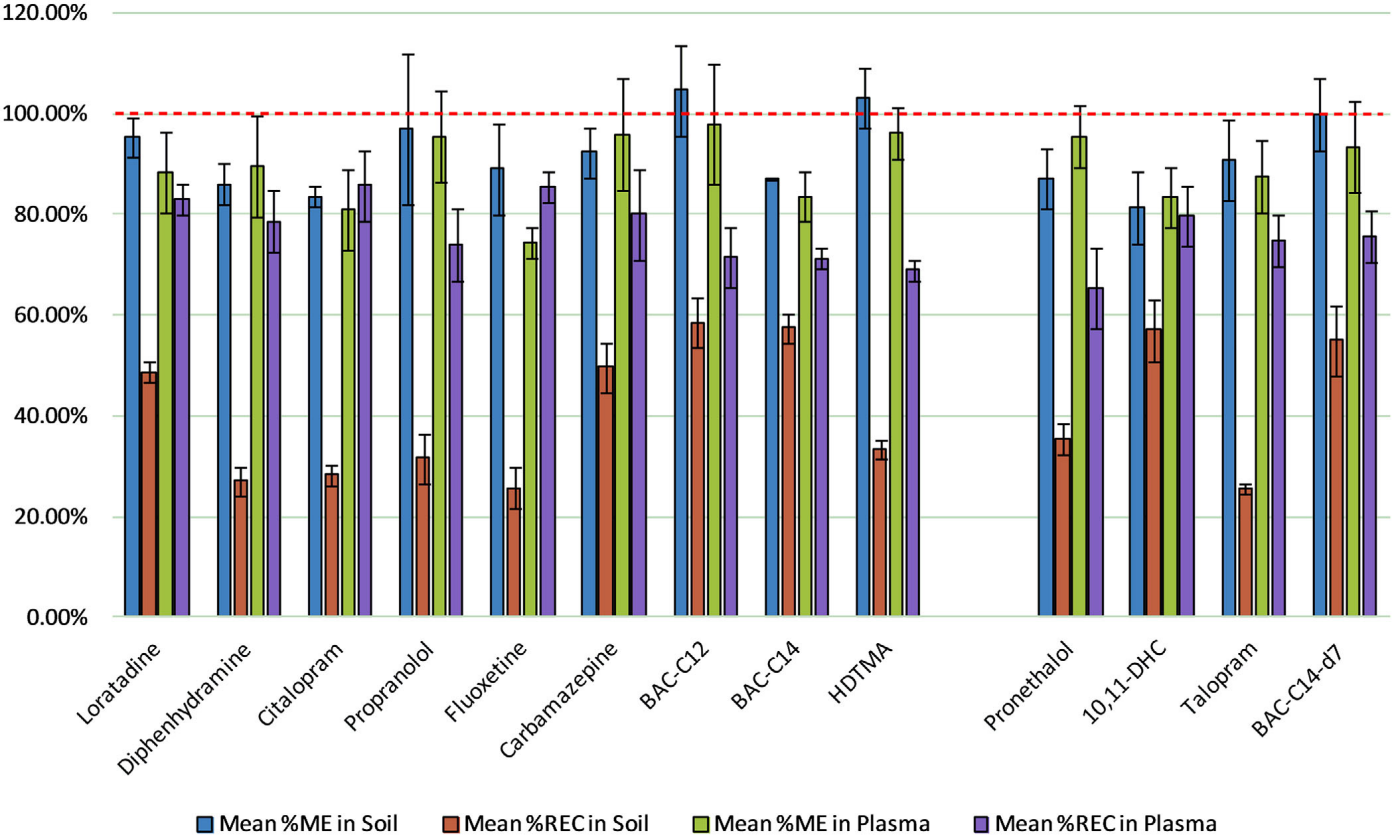
Comparison between the mean percentage matrix effects and recovery (with standard error bars) of acidic, neutral, basic and amphiphilic analytes calculated for the spiked soil and spiked blood plasma samples following micro‐QuEChERS extraction (n = 3). The dashed line indicated the zero matrix interference of the signal (i.e. 100% matrix effect)

The feasibility of this approach was based on preliminary work undertaken for a limited selection of pharmaceuticals (two) in biota,^17^ bisphenol A in urine^33^ and pesticides in tissues.^32^, ^41^ Typically, sample availability for environmental analyses are not a concern, however, for handling “hazardous” wastewater smaller sample sizes offer health and safety benefits by de‐risking the measurement of these materials. Also, despite these “proof‐of‐concept” studies, the capability of QuEChERS in extracting blood plasma for a broader range of medicinal compounds has remained unknown, even with a continued need for cleaner alternatives to “simple” clinical preparative methods. To assess the potential for extracting medicinal compounds from blood plasma and soil, a feasibility study of a “micro‐QuEChERS” extraction using this novel blend was carried out; this involved using lower sample volumes and extraction materials to provide a method that further reduces matrix effects (due to less matrix material) and operational cost. As expected, matrix effects (including enhancement) decreased for most analytes versus the larger‐scale QuEChERS approach with an overall median value of 90%ME, while maintaining the benefit of repeatability (RSD < 16%). However, despite the method showing largely repeatable recovery (i.e. ±20%) for each analyte the values were somewhat lower than anticipated (26‐59%), with erythromycin and acetaminophen undetectable following extraction regardless of sample matrix, indicating a decrease in extraction efficiency for these analytes (see Figure 2). Of the remaining analytes, the method performed very well for the amphiphilic class with similar recoveries to larger‐volume QuEChERS, showing significant promise for the scalability of our method in extracting these analytes in soil. However, the “micro‐QuEChERS” protocol showed the greatest promise when extracting the selected analytes from blood plasma; largely repeatable matrix effects and recovery were observed with 19% suppression and significantly higher recoveries for all analyte classes. This is particularly encouraging for clinical applications given quicker preparation methods are typically limited in the level of analyte recovery and matrix removal (e.g. lack of phospholipid extraction with protein precipitation), with the latter often resulting in significant matrix suppression (e.g. > 50%),^42^, ^43^, ^44^ with more limited sample availability requiring methods compatible with low(er) sample volumes.

This study indicates that the QuEChERS method can be robust in extracting a range of analytes (particularly amphiphilic species) from complex substances of high lipophilicity and organic content, such as soil and blood plasma, with flexibility in terms of sample volumes for the extraction. The method therefore offers promise for improving the handling of other environmental matrices of high organic content, such as wastewater effluent and sludge cake, or clinical samples (e.g. cerebrospinal fluid), potentially enabling the in‐process monitoring of pollutants or therapeutics, respectively, at reduced cost.

## 4 CONCLUDING REMARKS

Recognized sample preparation methods for analytes present in trace amounts within complex samples (e.g. environmental (semi) solids and biofluids) are typically multistep, time‐ and resource‐consuming extractions, with some used for environmental solids unsuitable for high‐throughput work, taking hours/sample. Unlike these protocols, QuEChERS has shown to be a time‐saving sample preparation method for some analyte classes but published work has not explicitly covered the breadth of acidic, basic, neutral, and amphiphilic analytes as a single protocol. To the best of the authors’ knowledge, this study is the first to address this need, showing the applicability of QuEChERS for extracting analytes of medicinal use (e.g. pharmaceuticals and biocides) from complex matrices as a single preparative method. Specifically, the protocol extracted analytes of variable lipophilicity/acidity from soil with largely repeatable matrix effects and recoveries, offering a quicker approach for environmental screening to inform future policy. To minimize sample handling risks and meet the demand for lower sample volumes (e.g. clinical applications), a reduced‐scale “micro‐QuEChERS” was also developed for soil and blood plasma. Interestingly, this showed reduced matrix enhancement, although analytes were recovered in lower amounts from soil than anticipated; however, and most pleasingly, all analytes were consistently extracted from blood plasma with improved matrix effects and recoveries, offering a promising method for clinical work. This novel sample preparation therefore not only provides a more efficient extraction for environmental matrices, with cost savings in terms of time and labor, but a quick “cleaner” alternative to “dilute and shoot” or protein precipitation for high‐throughput clinical work.

## CONFLICT OF INTEREST

The authors declare that they have no conflict of interest and ethical approval was not required for this work.

## Supporting information

Supporting Material
