# Supplementary material for: An investigation of the utility of QuEChERS for extracting acid, base, neutral and amphiphilic species from example environmental and clinical matrices
Source: Anal Sci Adv. 2020 Jun 5;1(3):152–60. doi: 10.1002/ansa.202000018 (PMC10989089; doi:10.1002/ansa.202000018)
Supplement: Supplementary file 1 — Supporting Material [file ANSA-1-152-s001.docx]

**An investigation of the utility of QuEChERS for extracting acid, base, neutral and amphiphilic species from example environmental and clinical matrices**

Rachel Townsend^1*^, Geertje van Keulen^2^, Claire Desbrow^3^. Amy Ruth Godfrey^1^

^1^Institute of Mass Spectrometry, Swansea University Medical School, Singleton Park, UK, SA2 8PP

^2^Institute of Life Science, Swansea University Medical School, Singleton Park, UK, SA2 8PP

^3^Biotage GB Limited, Distribution Way, Dyffryn Business Park, Ystrad Mynach, Hengoed, CF82 7TS

*Corresponding author:

Email: [rachel.townsend@swansea.ac.uk](mailto:748612@swansea.ac.uk)

**Equation S1:** Equation used to determine accuracy of the quantitative method(s).

$$\text{Accuracy (\%)=}\left[ \frac{\text{Measured concentration-theoretical concentration}}{\text{Theoretical concentration}} \right]\text{x 100}$$

**Equation S2:** Equation used to determine the precision of the quantitative method(s).

$$\text{Precision (\%)=}\left[ \frac{\text{Standard deviation of measured concentration}}{\text{Mean of measured concentration}} \right]\text{x 100}$$

**Equation S3:** Equation used to determine limit of detection of the analytes using the LC-MS method(s).

LOD = 3 x Standard deviation of the concentration of the blank sample

**Equation S4:** Equation used to determine the matrix effects of the sample preparation procedure.

$\text{Matrix Effects (\%)=}\left[ \frac{\text{Peak area of spike after extraction}}{\text{Peak area of standard}} \right]\text{x 100}$

**Equation S5:** Equation used to determine the matrix effects of the sample preparation procedure.

$\text{Recovery (\%)=}\left[ \frac{\text{Peak area of spike before extraction}}{\text{Peak area of spike after extraction}} \right]\text{x 100}$

**Equation S6:** Equation used to determine the matrix effects of the sample preparation procedure.

$\text{Process Efficiency (\%)=}\left[ \frac{\text{Peak area of spike before extraction}}{\text{Peak area of standard}} \right]\text{x 100 or }\left[ \frac{\text{ME x REC}}{\text{100}} \right]$

**Table S1:** Summary of physiochemical data and data acquisition parameters for each analyte. Assignment as acid, neutral, base has been made on chemical state at pH 7 given analyte pKa.

| **Analyte** | | **Molecular Formula** | **pKa** | **logP** | **Quadrupole** | | **Ion trap** | |
| --- | --- | --- | --- | --- | --- | --- | --- | --- |
|  |  |  |  |  | **Full mass scan (*m/z*)** | **Precursor *m/z* (SIM)** | **Full mass scan (*m/z*)** | **Precursor *m/z* (SRM fragment)** |
| **Acidic** | Diclofenac | C_14_H_11_Cl_2_NO_2_ | 4.4 | 4.06 | 125-775 | 296 | - | - |
|  | Loratadine | C_22_H_23_ClN_2_O_2_ | 4.7 | 5.94 |  | 383 | - | - |
| **Neutral** | Erythromycin | C_37_H_67_NO_13_ | 8.6 | 2.83 |  | 734 | - | - |
|  | Diphenhydramine | C_17_H_21_NO | 8.7 | 3.66 |  | 256 | - | - |
| **Basic** | Citalopram | C_20_H_21_FN_2_O | 9.4 | 2.51 |  | 325 | - | - |
|  | Propranolol | C_16_H_21_NO_2_ | 9.5 | 3.10 |  | 260 | - | - |
|  | Fluoxetine | C_17_H_18_F_3_NO | 9.6 | 4.09 |  | 310 | - | - |
|  | Acetaminophen | C_8_H_9_NO_2_ | 10.2 | 0.34 |  | 152 | - | - |
|  | Carbamazepine | C_15_H_12_N_2_O | 14.3 | 2.67 |  | 237 | - | - |
| **Amphiphilic** | BAC-C12 | C_21_H_38_N | - | 1.69 | - | - | 100-800 | 304 (212) |
|  | BAC-C14 | C_23_H_42_N | - | 2.55 | - | - |  | 332 (240) |
|  | HDTMA | C_19_H_42_N | - | 2.40 | - | - |  | 284 (n/a) |
| **Internal Standard** | Acetaminophen-(*methyl*-d_3_) | C_8_H_6_D_3_NO_2_ | - | 0.34 | 125-775 | 155 | - | - |
|  | Pronethalol | C_15_H_19_NO | - | 2.82 |  | 230 | - | - |
|  | 10,11-Dihydrocarbamazepine | C_15_H_14_N_2_O | - | 2.60 |  | 239 | - | - |
|  | Talopram | C_20_H_25_NO | - | 4.79 |  | 296 | - | - |
|  | BAC-C14-d_7_ | C_23_H_35_D_7_N | - |  | - | - | 100-800 | 339 (240) |

**Figure S1:** Chromatograms showing each of the analytes and internal standards.


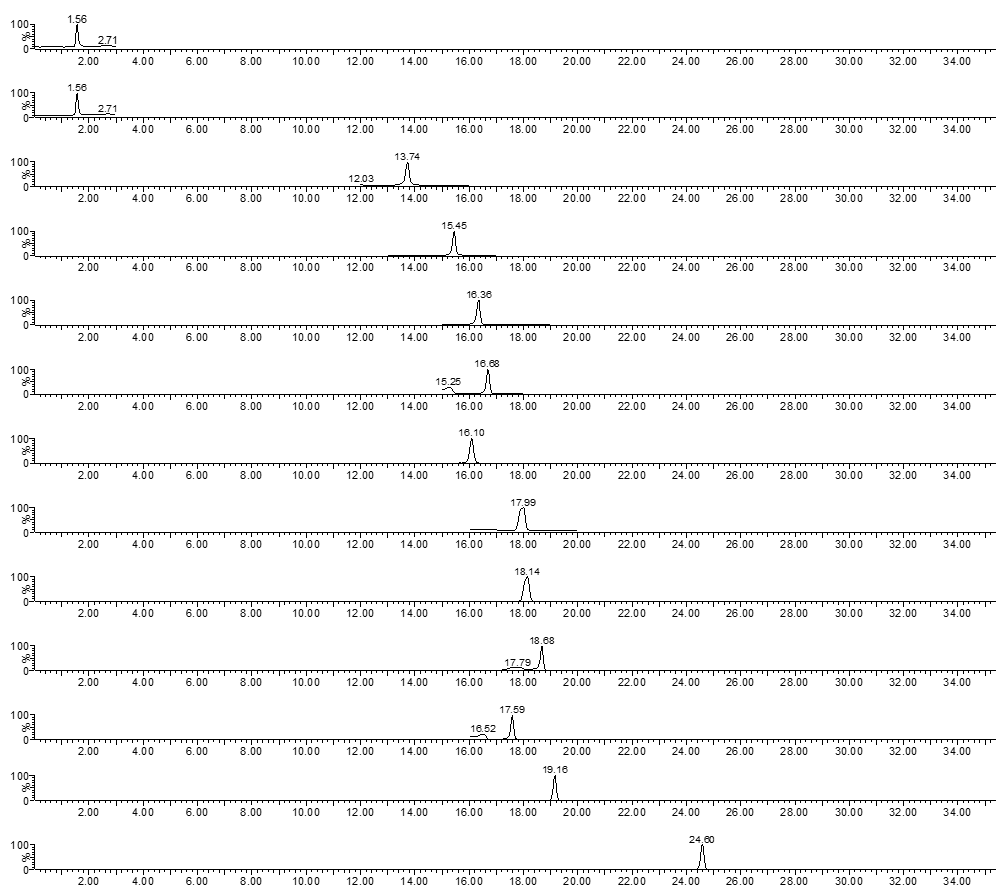


Acetaminophen

*m/z:* 152

Acetaminophen-(*methyl*-d_3_)

*m/z:* 155

Pronethalol

*m/z:* 230

Propranolol

*m/z:* 260

Diphenhydramine

*m/z:* 256

Citalopram

*m/z:* 325

Erythromycin

*m/z:* 734


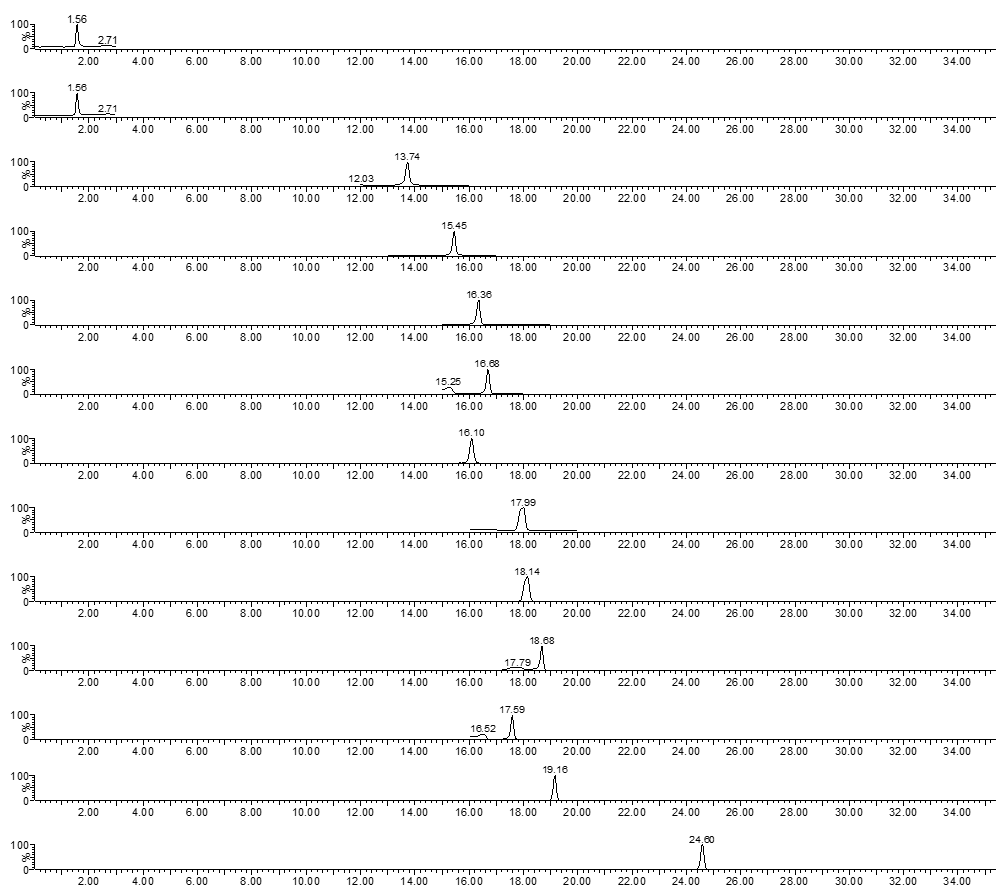


Carbamazepine

*m/z:* 237

10,11-Dihydrocarbamazepine

*m/z:* 239

Fluoxetine

*m/z:* 310

Talopram

*m/z:* 296

Loratadine

*m/z:* 383

Diclofenac

*m/z:* 296

BAC-C12

*m/z:* 304

BAC-C14

*m/z:* 332

HDTMA

*m/z:* 284

BAC-C14-d_7_

*m/z:* 339

**Table S2:** Chromatographic repeatability data for day 1 of the analytes and internal standards.

| **Analyte** | | **Replicate** | **Solvent Front (min)** | **Retention Time (min)** | **Adjusted Retention Time (min)** | **Mean RT** | **Mean Adjusted RT** | **Standard Deviation RT** | **Standard Deviation Adjusted RT** | **%RSD RT** | **%RSD Adjusted RT** |
| --- | --- | --- | --- | --- | --- | --- | --- | --- | --- | --- | --- |
| **Acidic** | Diclofenac  (*m/z* 296) | 1 | 1.15 | 24.88 | 23.73 | 24.67 | 23.55 | 0.10 | 0.09 | 0.40 | 0.38 |
|  |  | 2 | 1.12 | 24.75 | 23.63 |  |  |  |  |  |  |
|  |  | 3 | 1.12 | 24.75 | 23.63 |  |  |  |  |  |  |
|  |  | 4 | 1.09 | 24.67 | 23.58 |  |  |  |  |  |  |
|  |  | 5 | 1.12 | 24.65 | 23.53 |  |  |  |  |  |  |
|  |  | 6 | 1.12 | 24.62 | 23.50 |  |  |  |  |  |  |
|  |  | 7 | 1.12 | 24.62 | 23.50 |  |  |  |  |  |  |
|  |  | 8 | 1.12 | 24.59 | 23.47 |  |  |  |  |  |  |
|  |  | 9 | 1.09 | 24.57 | 23.48 |  |  |  |  |  |  |
|  |  | 10 | 1.12 | 24.57 | 23.45 |  |  |  |  |  |  |
|  | Loratadine  (*m/z* 383) | 1 | 1.15 | 19.48 | 18.33 | 19.25 | 18.13 | 0.11 | 0.10 | 0.55 | 0.55 |
|  |  | 2 | 1.12 | 19.33 | 18.21 |  |  |  |  |  |  |
|  |  | 3 | 1.12 | 19.31 | 18.19 |  |  |  |  |  |  |
|  |  | 4 | 1.09 | 19.26 | 18.17 |  |  |  |  |  |  |
|  |  | 5 | 1.12 | 19.27 | 18.15 |  |  |  |  |  |  |
|  |  | 6 | 1.12 | 19.17 | 18.05 |  |  |  |  |  |  |
|  |  | 7 | 1.12 | 19.19 | 18.07 |  |  |  |  |  |  |
|  |  | 8 | 1.12 | 19.12 | 18.00 |  |  |  |  |  |  |
|  |  | 9 | 1.09 | 19.19 | 18.10 |  |  |  |  |  |  |
|  |  | 10 | 1.12 | 19.16 | 18.04 |  |  |  |  |  |  |
| **Neutral** | Erythromycin  (*m/z* 734) | 1 | 1.15 | 16.52 | 15.37 | 16.23 | 15.11 | 0.12 | 0.11 | 0.76 | 0.75 |
|  |  | 2 | 1.12 | 16.32 | 15.20 |  |  |  |  |  |  |
|  |  | 3 | 1.12 | 16.29 | 15.17 |  |  |  |  |  |  |
|  |  | 4 | 1.09 | 16.19 | 15.10 |  |  |  |  |  |  |
|  |  | 5 | 1.12 | 16.2 | 15.08 |  |  |  |  |  |  |
|  |  | 6 | 1.12 | 16.16 | 15.04 |  |  |  |  |  |  |
|  |  | 7 | 1.12 | 16.19 | 15.07 |  |  |  |  |  |  |
|  |  | 8 | 1.12 | 16.14 | 15.02 |  |  |  |  |  |  |
|  |  | 9 | 1.09 | 16.14 | 15.05 |  |  |  |  |  |  |
|  |  | 10 | 1.12 | 16.1 | 14.98 |  |  |  |  |  |  |
|  | Diphenhydramine  (*m/z* 256) | 1 | 1.15 | 16.82 | 15.67 | 16.47 | 15.35 | 0.14 | 0.13 | 0.87 | 0.86 |
|  |  | 2 | 1.12 | 16.58 | 15.46 |  |  |  |  |  |  |
|  |  | 3 | 1.12 | 16.55 | 15.43 |  |  |  |  |  |  |
|  |  | 4 | 1.09 | 16.4 | 15.31 |  |  |  |  |  |  |
|  |  | 5 | 1.12 | 16.42 | 15.30 |  |  |  |  |  |  |
|  |  | 6 | 1.12 | 16.42 | 15.30 |  |  |  |  |  |  |
|  |  | 7 | 1.12 | 16.4 | 15.28 |  |  |  |  |  |  |
|  |  | 8 | 1.12 | 16.36 | 15.24 |  |  |  |  |  |  |
|  |  | 9 | 1.09 | 16.4 | 15.31 |  |  |  |  |  |  |
|  |  | 10 | 1.12 | 16.36 | 15.24 |  |  |  |  |  |  |
| **Basic** | Citalopram  (*m/*z 325) | 1 | 1.15 | 17.13 | 15.98 | 16.80 | 15.69 | 0.13 | 0.12 | 0.79 | 0.78 |
|  |  | 2 | 1.12 | 16.9 | 15.78 |  |  |  |  |  |  |
|  |  | 3 | 1.12 | 16.87 | 15.75 |  |  |  |  |  |  |
|  |  | 4 | 1.09 | 16.77 | 15.68 |  |  |  |  |  |  |
|  |  | 5 | 1.12 | 16.74 | 15.62 |  |  |  |  |  |  |
|  |  | 6 | 1.12 | 16.74 | 15.62 |  |  |  |  |  |  |
|  |  | 7 | 1.12 | 16.77 | 15.65 |  |  |  |  |  |  |
|  |  | 8 | 1.12 | 16.72 | 15.60 |  |  |  |  |  |  |
|  |  | 9 | 1.09 | 16.72 | 15.63 |  |  |  |  |  |  |
|  |  | 10 | 1.12 | 16.68 | 15.56 |  |  |  |  |  |  |
|  | Propranolol  (*m/z* 260) | 1 | 1.15 | 15.98 | 14.83 | 15.61 | 14.49 | 0.16 | 0.15 | 1.00 | 1.00 |
|  |  | 2 | 1.12 | 15.72 | 14.60 |  |  |  |  |  |  |
|  |  | 3 | 1.12 | 15.71 | 14.59 |  |  |  |  |  |  |
|  |  | 4 | 1.09 | 15.55 | 14.46 |  |  |  |  |  |  |
|  |  | 5 | 1.12 | 15.54 | 14.42 |  |  |  |  |  |  |
|  |  | 6 | 1.12 | 15.54 | 14.42 |  |  |  |  |  |  |
|  |  | 7 | 1.12 | 15.55 | 14.43 |  |  |  |  |  |  |
|  |  | 8 | 1.12 | 15.48 | 14.36 |  |  |  |  |  |  |
|  |  | 9 | 1.09 | 15.52 | 14.43 |  |  |  |  |  |  |
|  |  | 10 | 1.12 | 15.48 | 14.36 |  |  |  |  |  |  |
|  | Fluoxetine  (*m/z* 310) | 1 | 1.15 | 18.99 | 17.84 | 18.76 | 17.65 | 0.11 | 0.10 | 0.58 | 0.56 |
|  |  | 2 | 1.12 | 18.87 | 17.75 |  |  |  |  |  |  |
|  |  | 3 | 1.12 | 18.84 | 17.72 |  |  |  |  |  |  |
|  |  | 4 | 1.09 | 18.75 | 17.66 |  |  |  |  |  |  |
|  |  | 5 | 1.12 | 18.76 | 17.64 |  |  |  |  |  |  |
|  |  | 6 | 1.12 | 18.69 | 17.57 |  |  |  |  |  |  |
|  |  | 7 | 1.12 | 18.72 | 17.60 |  |  |  |  |  |  |
|  |  | 8 | 1.12 | 18.64 | 17.52 |  |  |  |  |  |  |
|  |  | 9 | 1.09 | 18.68 | 17.59 |  |  |  |  |  |  |
|  |  | 10 | 1.12 | 18.68 | 17.56 |  |  |  |  |  |  |
|  | Acetaminophen  (*m/z* 152) | 1 | 1.15 | 1.59 | 0.44 | 1.56 | 0.45 | 0.01 | 0.01 | 0.61 | 2.84 |
|  |  | 2 | 1.12 | 1.56 | 0.44 |  |  |  |  |  |  |
|  |  | 3 | 1.12 | 1.56 | 0.44 |  |  |  |  |  |  |
|  |  | 4 | 1.09 | 1.56 | 0.47 |  |  |  |  |  |  |
|  |  | 5 | 1.12 | 1.56 | 0.44 |  |  |  |  |  |  |
|  |  | 6 | 1.12 | 1.56 | 0.44 |  |  |  |  |  |  |
|  |  | 7 | 1.12 | 1.56 | 0.44 |  |  |  |  |  |  |
|  |  | 8 | 1.12 | 1.56 | 0.44 |  |  |  |  |  |  |
|  |  | 9 | 1.09 | 1.56 | 0.47 |  |  |  |  |  |  |
|  |  | 10 | 1.12 | 1.56 | 0.44 |  |  |  |  |  |  |
|  | Carbamazepine  (*m/z* 237) | 1 | 1.15 | 18.42 | 17.27 | 18.11 | 16.99 | 0.13 | 0.12 | 0.74 | 0.74 |
|  |  | 2 | 1.12 | 18.22 | 17.10 |  |  |  |  |  |  |
|  |  | 3 | 1.12 | 18.19 | 17.07 |  |  |  |  |  |  |
|  |  | 4 | 1.09 | 18.07 | 16.98 |  |  |  |  |  |  |
|  |  | 5 | 1.12 | 18.08 | 16.96 |  |  |  |  |  |  |
|  |  | 6 | 1.12 | 18.04 | 16.92 |  |  |  |  |  |  |
|  |  | 7 | 1.12 | 18.03 | 16.91 |  |  |  |  |  |  |
|  |  | 8 | 1.12 | 17.99 | 16.87 |  |  |  |  |  |  |
|  |  | 9 | 1.09 | 18.03 | 16.94 |  |  |  |  |  |  |
|  |  | 10 | 1.12 | 17.99 | 16.87 |  |  |  |  |  |  |
| **Amphiphilic** | BAC-12  (*m/z* 304) | 1 | 1.10 | 24.20 | 23.10 | 24.20 | 23.11 | 0.04 | 0.05 | 0.16 | 0.23 |
|  |  | 2 | 1.06 | 24.24 | 23.18 |  |  |  |  |  |  |
|  |  | 3 | 1.10 | 24.22 | 23.12 |  |  |  |  |  |  |
|  |  | 4 | 1.11 | 24.20 | 23.09 |  |  |  |  |  |  |
|  |  | 5 | 1.06 | 24.20 | 23.14 |  |  |  |  |  |  |
|  |  | 6 | 1.11 | 24.14 | 23.03 |  |  |  |  |  |  |
|  |  | 7 | 1.11 | 24.15 | 23.04 |  |  |  |  |  |  |
|  |  | 8 | 1.10 | 24.18 | 23.08 |  |  |  |  |  |  |
|  |  | 9 | 1.06 | 24.17 | 23.11 |  |  |  |  |  |  |
|  |  | 10 | 1.07 | 24.26 | 23.19 |  |  |  |  |  |  |
|  | BAC-14  (*m/z* 332) | 1 | 1.10 | 26.42 | 25.32 | 26.42 | 25.33 | 0.03 | 0.03 | 0.12 | 0.13 |
|  |  | 2 | 1.06 | 26.43 | 25.37 |  |  |  |  |  |  |
|  |  | 3 | 1.10 | 26.44 | 25.34 |  |  |  |  |  |  |
|  |  | 4 | 1.11 | 26.47 | 25.36 |  |  |  |  |  |  |
|  |  | 5 | 1.06 | 26.41 | 25.35 |  |  |  |  |  |  |
|  |  | 6 | 1.11 | 26.36 | 25.25 |  |  |  |  |  |  |
|  |  | 7 | 1.11 | 26.43 | 25.32 |  |  |  |  |  |  |
|  |  | 8 | 1.10 | 26.43 | 25.33 |  |  |  |  |  |  |
|  |  | 9 | 1.06 | 26.37 | 25.31 |  |  |  |  |  |  |
|  |  | 10 | 1.07 | 26.42 | 25.35 |  |  |  |  |  |  |
|  | HDTMA  (*m/z* 284) | 1 | 1.10 | 26.33 | 25.23 | 26.31 | 25.22 | 0.03 | 0.04 | 0.10 | 0.16 |
|  |  | 2 | 1.06 | 26.34 | 25.28 |  |  |  |  |  |  |
|  |  | 3 | 1.10 | 26.29 | 25.19 |  |  |  |  |  |  |
|  |  | 4 | 1.11 | 26.32 | 25.21 |  |  |  |  |  |  |
|  |  | 5 | 1.06 | 26.33 | 25.27 |  |  |  |  |  |  |
|  |  | 6 | 1.11 | 26.27 | 25.16 |  |  |  |  |  |  |
|  |  | 7 | 1.11 | 26.29 | 25.18 |  |  |  |  |  |  |
|  |  | 8 | 1.10 | 26.28 | 25.18 |  |  |  |  |  |  |
|  |  | 9 | 1.06 | 26.28 | 25.22 |  |  |  |  |  |  |
|  |  | 10 | 1.07 | 26.33 | 25.26 |  |  |  |  |  |  |
| **Internal Standards** | Acetaminophen-(methyl-d3)  (*m/z* 155) | 1 | 1.15 | 1.59 | 0.44 | 1.56 | 0.45 | 0.01 | 0.01 | 0.61 | 2.84 |
|  |  | 2 | 1.12 | 1.56 | 0.44 |  |  |  |  |  |  |
|  |  | 3 | 1.12 | 1.56 | 0.44 |  |  |  |  |  |  |
|  |  | 4 | 1.09 | 1.56 | 0.47 |  |  |  |  |  |  |
|  |  | 5 | 1.12 | 1.56 | 0.44 |  |  |  |  |  |  |
|  |  | 6 | 1.12 | 1.56 | 0.44 |  |  |  |  |  |  |
|  |  | 7 | 1.12 | 1.56 | 0.44 |  |  |  |  |  |  |
|  |  | 8 | 1.12 | 1.56 | 0.44 |  |  |  |  |  |  |
|  |  | 9 | 1.09 | 1.56 | 0.47 |  |  |  |  |  |  |
|  |  | 10 | 1.12 | 1.56 | 0.44 |  |  |  |  |  |  |
|  | Pronethalol  (*m/z* 230) | 1 | 1.15 | 14.45 | 13.30 | 13.93 | 12.81 | 0.21 | 0.20 | 1.54 | 1.59 |
|  |  | 2 | 1.12 | 14.09 | 12.97 |  |  |  |  |  |  |
|  |  | 3 | 1.12 | 14.05 | 12.93 |  |  |  |  |  |  |
|  |  | 4 | 1.09 | 13.90 | 12.81 |  |  |  |  |  |  |
|  |  | 5 | 1.12 | 13.83 | 12.71 |  |  |  |  |  |  |
|  |  | 6 | 1.12 | 13.83 | 12.71 |  |  |  |  |  |  |
|  |  | 7 | 1.12 | 13.83 | 12.71 |  |  |  |  |  |  |
|  |  | 8 | 1.12 | 13.76 | 12.64 |  |  |  |  |  |  |
|  |  | 9 | 1.09 | 13.81 | 12.72 |  |  |  |  |  |  |
|  |  | 10 | 1.12 | 13.76 | 12.64 |  |  |  |  |  |  |
|  | DHC  (*m/z* 239) | 1 | 1.15 | 18.57 | 17.42 | 18.26 | 17.14 | 0.13 | 0.13 | 0.73 | 0.73 |
|  |  | 2 | 1.12 | 18.37 | 17.25 |  |  |  |  |  |  |
|  |  | 3 | 1.12 | 18.34 | 17.22 |  |  |  |  |  |  |
|  |  | 4 | 1.09 | 18.26 | 17.17 |  |  |  |  |  |  |
|  |  | 5 | 1.12 | 18.23 | 17.11 |  |  |  |  |  |  |
|  |  | 6 | 1.12 | 18.19 | 17.07 |  |  |  |  |  |  |
|  |  | 7 | 1.12 | 18.18 | 17.06 |  |  |  |  |  |  |
|  |  | 8 | 1.12 | 18.14 | 17.02 |  |  |  |  |  |  |
|  |  | 9 | 1.09 | 18.18 | 17.09 |  |  |  |  |  |  |
|  |  | 10 | 1.12 | 18.14 | 17.02 |  |  |  |  |  |  |
|  | Talopram  (*m/z* 296) | 1 | 1.15 | 17.97 | 16.82 | 17.69 | 16.57 | 0.12 | 0.11 | 0.69 | 0.68 |
|  |  | 2 | 1.12 | 17.79 | 16.67 |  |  |  |  |  |  |
|  |  | 3 | 1.12 | 17.77 | 16.65 |  |  |  |  |  |  |
|  |  | 4 | 1.09 | 17.67 | 16.58 |  |  |  |  |  |  |
|  |  | 5 | 1.12 | 17.64 | 16.52 |  |  |  |  |  |  |
|  |  | 6 | 1.12 | 17.60 | 16.48 |  |  |  |  |  |  |
|  |  | 7 | 1.12 | 17.63 | 16.51 |  |  |  |  |  |  |
|  |  | 8 | 1.12 | 17.59 | 16.47 |  |  |  |  |  |  |
|  |  | 9 | 1.09 | 17.63 | 16.54 |  |  |  |  |  |  |
|  |  | 10 | 1.12 | 17.59 | 16.47 |  |  |  |  |  |  |
|  | BAC-C14-d7  (*m/z* 339) | 1 | 1.10 | 26.39 | 25.29 | 26.38 | 25.29 | 0.04 | 0.05 | 0.16 | 0.21 |
|  |  | 2 | 1.06 | 26.45 | 25.39 |  |  |  |  |  |  |
|  |  | 3 | 1.10 | 26.41 | 25.31 |  |  |  |  |  |  |
|  |  | 4 | 1.11 | 26.44 | 25.33 |  |  |  |  |  |  |
|  |  | 5 | 1.06 | 26.38 | 25.32 |  |  |  |  |  |  |
|  |  | 6 | 1.11 | 26.33 | 25.22 |  |  |  |  |  |  |
|  |  | 7 | 1.11 | 26.34 | 25.23 |  |  |  |  |  |  |
|  |  | 8 | 1.10 | 26.34 | 25.24 |  |  |  |  |  |  |
|  |  | 9 | 1.06 | 26.34 | 25.28 |  |  |  |  |  |  |
|  |  | 10 | 1.07 | 26.39 | 25.32 |  |  |  |  |  |  |

**Table S2:** Chromatographic repeatability data for day 2 of the analytes and internal standards.

| **Analyte** | | **Replicate** | **Solvent Front (min)** | **Retention Time (min)** | **Adjusted Retention Time (min)** | **Mean RT** | **Mean Adjusted RT** | **Standard Deviation RT** | **Standard Deviation Adjusted RT** | **%RSD RT** | **%RSD Adjusted RT** |
| --- | --- | --- | --- | --- | --- | --- | --- | --- | --- | --- | --- |
| **Acidic** | Diclofenac  (*m/z* 296) | 11 | 1.09 | 24.52 | 23.43 | 24.56 | 23.47 | 0.04 | 0.05 | 0.16 | 0.23 |
|  |  | 12 | 1.09 | 24.59 | 23.50 |  |  |  |  |  |  |
|  |  | 13 | 1.09 | 24.55 | 23.46 |  |  |  |  |  |  |
|  |  | 14 | 1.07 | 24.62 | 23.55 |  |  |  |  |  |  |
|  |  | 15 | 1.12 | 24.52 | 23.40 |  |  |  |  |  |  |
|  |  | 16 | 1.12 | 24.57 | 23.45 |  |  |  |  |  |  |
|  | Loratadine  (*m/z* 383) | 11 | 1.09 | 19.16 | 18.07 | 19.14 | 18.05 | 0.02 | 0.03 | 0.13 | 0.18 |
|  |  | 12 | 1.09 | 19.15 | 18.06 |  |  |  |  |  |  |
|  |  | 13 | 1.09 | 19.10 | 18.01 |  |  |  |  |  |  |
|  |  | 14 | 1.07 | 19.16 | 18.09 |  |  |  |  |  |  |
|  |  | 15 | 1.12 | 19.13 | 18.01 |  |  |  |  |  |  |
|  |  | 16 | 1.12 | 19.16 | 18.04 |  |  |  |  |  |  |
| **Neutral** | Erythromycin  (*m/z* 734) | 11 | 1.09 | 16.1 | 15.01 | 16.10 | 15.01 | 0.02 | 0.03 | 0.14 | 0.23 |
|  |  | 12 | 1.09 | 16.1 | 15.01 |  |  |  |  |  |  |
|  |  | 13 | 1.09 | 16.07 | 14.98 |  |  |  |  |  |  |
|  |  | 14 | 1.07 | 16.14 | 15.07 |  |  |  |  |  |  |
|  |  | 15 | 1.12 | 16.11 | 14.99 |  |  |  |  |  |  |
|  |  | 16 | 1.12 | 16.1 | 14.98 |  |  |  |  |  |  |
|  | Diphenhydramine  (*m/z* 256) | 11 | 1.09 | 16.32 | 15.23 | 16.33 | 15.23 | 0.03 | 0.04 | 0.16 | 0.29 |
|  |  | 12 | 1.09 | 16.36 | 15.27 |  |  |  |  |  |  |
|  |  | 13 | 1.09 | 16.33 | 15.24 |  |  |  |  |  |  |
|  |  | 14 | 1.07 | 16.36 | 15.29 |  |  |  |  |  |  |
|  |  | 15 | 1.12 | 16.29 | 15.17 |  |  |  |  |  |  |
|  |  | 16 | 1.12 | 16.32 | 15.20 |  |  |  |  |  |  |
| **Basic** | Citalopram  (*m/*z 325) | 11 | 1.09 | 16.68 | 15.59 | 16.67 | 15.57 | 0.04 | 0.06 | 0.24 | 0.36 |
|  |  | 12 | 1.09 | 16.68 | 15.59 |  |  |  |  |  |  |
|  |  | 13 | 1.09 | 16.65 | 15.56 |  |  |  |  |  |  |
|  |  | 14 | 1.07 | 16.72 | 15.65 |  |  |  |  |  |  |
|  |  | 15 | 1.12 | 16.6 | 15.48 |  |  |  |  |  |  |
|  |  | 16 | 1.12 | 16.68 | 15.56 |  |  |  |  |  |  |
|  | Propranolol  (*m/z* 260) | 11 | 1.09 | 15.45 | 14.36 | 15.44 | 14.35 | 0.03 | 0.04 | 0.17 | 0.28 |
|  |  | 12 | 1.09 | 15.48 | 14.39 |  |  |  |  |  |  |
|  |  | 13 | 1.09 | 15.43 | 14.34 |  |  |  |  |  |  |
|  |  | 14 | 1.07 | 15.45 | 14.38 |  |  |  |  |  |  |
|  |  | 15 | 1.12 | 15.40 | 14.28 |  |  |  |  |  |  |
|  |  | 16 | 1.12 | 15.45 | 14.33 |  |  |  |  |  |  |
|  | Fluoxetine  (*m/z* 310) | 11 | 1.09 | 18.64 | 17.55 | 18.65 | 17.55 | 0.03 | 0.04 | 0.18 | 0.24 |
|  |  | 12 | 1.09 | 18.67 | 17.58 |  |  |  |  |  |  |
|  |  | 13 | 1.09 | 18.61 | 17.52 |  |  |  |  |  |  |
|  |  | 14 | 1.07 | 18.68 | 17.61 |  |  |  |  |  |  |
|  |  | 15 | 1.12 | 18.61 | 17.49 |  |  |  |  |  |  |
|  |  | 16 | 1.12 | 18.68 | 17.56 |  |  |  |  |  |  |
|  | Acetaminophen  (*m/z* 152) | 11 | 1.09 | 1.56 | 0.47 | 1.56 | 0.46 | 0.01 | 0.03 | 0.52 | 5.50 |
|  |  | 12 | 1.09 | 1.56 | 0.47 |  |  |  |  |  |  |
|  |  | 13 | 1.09 | 1.56 | 0.47 |  |  |  |  |  |  |
|  |  | 14 | 1.07 | 1.56 | 0.49 |  |  |  |  |  |  |
|  |  | 15 | 1.12 | 1.54 | 0.42 |  |  |  |  |  |  |
|  |  | 16 | 1.12 | 1.56 | 0.44 |  |  |  |  |  |  |
|  | Carbamazepine  (*m/z* 237) | 11 | 1.09 | 17.99 | 16.90 | 17.97 | 16.88 | 0.04 | 0.05 | 0.23 | 0.31 |
|  |  | 12 | 1.09 | 18.02 | 16.93 |  |  |  |  |  |  |
|  |  | 13 | 1.09 | 17.92 | 16.83 |  |  |  |  |  |  |
|  |  | 14 | 1.07 | 17.99 | 16.92 |  |  |  |  |  |  |
|  |  | 15 | 1.12 | 17.92 | 16.80 |  |  |  |  |  |  |
|  |  | 16 | 1.12 | 17.99 | 16.87 |  |  |  |  |  |  |
| **Amphiphilic** | BAC-12  (*m/z* 304) | 11 | 1.11 | 24.10 | 22.99 | 24.13 | 23.03 | 0.02 | 0.03 | 0.09 | 0.12 |
|  |  | 12 | 1.07 | 24.13 | 23.06 |  |  |  |  |  |  |
|  |  | 13 | 1.10 | 24.13 | 23.03 |  |  |  |  |  |  |
|  |  | 14 | 1.10 | 24.11 | 23.01 |  |  |  |  |  |  |
|  |  | 15 | 1.10 | 24.12 | 23.02 |  |  |  |  |  |  |
|  |  | 16 | 1.10 | 24.16 | 23.06 |  |  |  |  |  |  |
|  | BAC-14  (*m/z* 332) | 11 | 1.11 | 26.35 | 25.24 | 26.37 | 25.27 | 0.03 | 0.03 | 0.12 | 0.13 |
|  |  | 12 | 1.07 | 26.35 | 25.28 |  |  |  |  |  |  |
|  |  | 13 | 1.10 | 26.35 | 25.25 |  |  |  |  |  |  |
|  |  | 14 | 1.10 | 26.35 | 25.25 |  |  |  |  |  |  |
|  |  | 15 | 1.10 | 26.43 | 25.33 |  |  |  |  |  |  |
|  |  | 16 | 1.10 | 26.38 | 25.28 |  |  |  |  |  |  |
|  | HDTMA  (*m/z* 284) | 11 | 1.11 | 26.21 | 25.10 | 26.22 | 25.12 | 0.01 | 0.02 | 0.05 | 0.07 |
|  |  | 12 | 1.07 | 26.21 | 25.14 |  |  |  |  |  |  |
|  |  | 13 | 1.10 | 26.21 | 25.11 |  |  |  |  |  |  |
|  |  | 14 | 1.10 | 26.21 | 25.11 |  |  |  |  |  |  |
|  |  | 15 | 1.10 | 26.23 | 25.13 |  |  |  |  |  |  |
|  |  | 16 | 1.10 | 26.24 | 25.14 |  |  |  |  |  |  |
| **Internal Standard** | Acetaminophen-(methyl-d3)  (*m/z* 155) | 11 | 1.09 | 1.56 | 0.47 | 1.56 | 0.46 | 0.00 | 0.02 | 0.00 | 4.24 |
|  |  | 12 | 1.09 | 1.56 | 0.47 |  |  |  |  |  |  |
|  |  | 13 | 1.09 | 1.56 | 0.47 |  |  |  |  |  |  |
|  |  | 14 | 1.07 | 1.56 | 0.49 |  |  |  |  |  |  |
|  |  | 15 | 1.12 | 1.56 | 0.44 |  |  |  |  |  |  |
|  |  | 16 | 1.12 | 1.56 | 0.44 |  |  |  |  |  |  |
|  | Pronethalol  (*m/z* 230) | 11 | 1.09 | 13.74 | 12.65 | 13.74 | 12.65 | 0.04 | 0.05 | 0.28 | 0.40 |
|  |  | 12 | 1.09 | 13.81 | 12.72 |  |  |  |  |  |  |
|  |  | 13 | 1.09 | 13.74 | 12.65 |  |  |  |  |  |  |
|  |  | 14 | 1.07 | 13.74 | 12.67 |  |  |  |  |  |  |
|  |  | 15 | 1.12 | 13.69 | 12.57 |  |  |  |  |  |  |
|  |  | 16 | 1.12 | 13.74 | 12.62 |  |  |  |  |  |  |
|  | DHC  (*m/z* 239) | 11 | 1.09 | 18.14 | 17.05 | 18.13 | 17.03 | 0.02 | 0.04 | 0.13 | 0.23 |
|  |  | 12 | 1.09 | 18.14 | 17.05 |  |  |  |  |  |  |
|  |  | 13 | 1.09 | 18.12 | 17.03 |  |  |  |  |  |  |
|  |  | 14 | 1.07 | 18.14 | 17.07 |  |  |  |  |  |  |
|  |  | 15 | 1.12 | 18.08 | 16.96 |  |  |  |  |  |  |
|  |  | 16 | 1.12 | 18.14 | 17.02 |  |  |  |  |  |  |
|  | Talopram  (*m/z* 296) | 11 | 1.09 | 17.59 | 16.50 | 17.57 | 16.48 | 0.03 | 0.04 | 0.16 | 0.26 |
|  |  | 12 | 1.09 | 17.59 | 16.50 |  |  |  |  |  |  |
|  |  | 13 | 1.09 | 17.56 | 16.47 |  |  |  |  |  |  |
|  |  | 14 | 1.07 | 17.59 | 16.52 |  |  |  |  |  |  |
|  |  | 15 | 1.12 | 17.52 | 16.40 |  |  |  |  |  |  |
|  |  | 16 | 1.12 | 17.59 | 16.47 |  |  |  |  |  |  |
|  | BAC-C14-d7  (*m/z* 339) | 11 | 1.11 | 26.32 | 25.21 | 26.32 | 25.22 | 0.03 | 0.03 | 0.10 | 0.12 |
|  |  | 12 | 1.07 | 26.32 | 25.25 |  |  |  |  |  |  |
|  |  | 13 | 1.10 | 26.32 | 25.22 |  |  |  |  |  |  |
|  |  | 14 | 1.10 | 26.27 | 25.17 |  |  |  |  |  |  |
|  |  | 15 | 1.10 | 26.34 | 25.24 |  |  |  |  |  |  |
|  |  | 16 | 1.10 | 26.35 | 25.25 |  |  |  |  |  |  |

**Table S3:** Summary of chromatographic reproducibility of adjusted retention times for each analyte (day 1: n=10, day 2: n=6 for acid, neutral, base, and n=7 for amphiphilic). Chromatographic repeatability is represented by %RSD and reproducibility by two-tailed F- test; F-stat were F_(9,5)_ 6.68^a)^, F_(5,9)_ 4.48^b)^ for acidic, neutral, basic analytes and F_(9,6)_ 5.523^c)^, F_(6,9)_ 4.320^d)^ for amphiphilic analytes.

| **Analyte** | | **Chromatographic Stability** | | |
| --- | --- | --- | --- | --- |
|  |  | **Repeatability** | | **Reproducibility** |
|  |  | **Day 1** | **Day 2** |  |
| **Acidic** | Diclofenac | 0.38 | 0.23 | 2.86 ^a)^ |
|  | Loratadine | 0.55 | 0.18 | 9.16 ^a)^ |
| **Neutral** | Erythromycin | 0.75 | 0.23 | 11.18 ^a)^ |
|  | Diphenhydramine | 0.86 | 0.29 | 9.02 ^a)^ |
| **Basic** | Citalopram | 0.78 | 0.36 | 4.87 ^a)^ |
|  | Propranolol | 1.00 | 0.28 | 13.26 ^a)^ |
|  | Fluoxetine | 0.56 | 0.24 | 5.41^a)^ |
|  | Acetaminophen | 2.84 | 5.50 | 4.00 ^b)^ |
|  | Carbamazepine | 0.74 | 0.31 | 5.85 ^a)^ |
| **Amphiphilic** | BAC-C12 | 0.23 | 0.24 | 1.13 ^d)^ |
|  | BAC-C14 | 0.13 | 0.19 | 1.99 ^d)^ |
|  | HDTMA | 0.16 | 0.17 | 1.11 ^d)^ |
| **Internal Standard** | Acetaminophen-(*methyl*-d_3_) | 2.84 | 4.24 | 2.42^b)^ |
|  | Pronethalol | 1.59 | 0.40 | 16.66 ^a)^ |
|  | 10,11-Dihydrocarbamazepine | 0.73 | 0.23 | 10.60 ^a)^ |
|  | Talopram | 0.68 | 0.26 | 7.10 ^a)^ |
|  | BAC-C14-d_7_ | 0.21 | 0.12 | 1.01^c)^ |

**Table S4:** Injection repeatability data for day 2 of the analytes and internal standards.

| **Analyte** | | **Peak Area** | | **F_(9,5)_ 6.681** | **F_(5,9)_ 4.484** |
| --- | --- | --- | --- | --- | --- |
|  |  | **%CV Day 1** | **%CV Day 2** | **F-Test** | **F-Test** |
| Acidic | Diclofenac | 1.38 | 0.70 |  | 4.22 |
|  | Loratadine | 3.26 | 1.40 | 6.25 |  |
| Neutral | Erythromycin | 1.34 | 1.08 | 1.74 |  |
|  | Diphenhydramine | 1.80 | 1.65 | 1.15 |  |
| Basic | Citalopram | 1.59 | 1.85 |  | 1.29 |
|  | Propranolol | 1.34 | 0.77 | 3.08 |  |
|  | Fluoxetine | 1.45 | 1.01 |  | 2.20 |
|  | Acetaminophen | 1.38 | 1.16 | 1.54 |  |
|  | Carbamazepine | 4.52 | 1.59 | 9.19 |  |
| Amphiphilic | BAC-C12 | 5.36 | 5.63 |  | 1.16 |
|  | BAC-C14 | 8.75 | 8.63 |  | 1.09 |
|  | HDTMA | 4.64 | 6.37 |  | 1.96 |
| Internal Standards | Acet-d3 | 6.85 | 5.03 | 1.94 |  |
|  | Pronethalol | 2.33 | 1.13 | 4.39 |  |
|  | 10,11-DHC | 1.86 | 0.55 | 11.75 |  |
|  | Talopram | 2.15 | 2.99 |  | 1.87 |
|  | BAC-C14-d7 | 11.89 | 7.05 | 3.02 |  |

**Table S5:** Summary of heteroscedasticity data for each analyte, including the calibration equation and total percentage relative error (%RE) for equal (linear) and weighted regression functions.

| **Analyte** | **Internal Standard (IS)** | **Calibration Range (ng/mL)** | **Calibration Equations (R^2^)** | | **Total Relative Error (%RErr)** | |
| --- | --- | --- | --- | --- | --- | --- |
|  |  |  | **Linear (w = 1)** | **Weighted (w = 1/x)** | **Linear (w = 1)** | **Weighted (w = 1/x)** |
| Diclofenac | Talopram | 1-400 | y = 0.0017x + 0.0050 (0.999) | y = 0.0017x + 0.0040  (0.998) | -289.94 | -33.10 |
| Loratadine | 10,11-Dihydrochloride |  | y = 0.0037x + 0.0012 (0.997) | y = 0.0037x + 0.0002  (0.998) | -108.74 | -2.37 |
| Erythromycin | 10,11-Dihydrochloride |  | y = 0.00044x + 0.00041 (0.999) | y = 0.00044x + 0.00013  (0.999) | -259.03 | -4.35 |
| Diphenhydramine | Talopram |  | y = 0.0099x + 0.026 (0.999) | y = 0.010x + 0.0069  (0.999) | -626.16 | 4.50 |
| Citalopram | Talopram |  | y = 0.0076x + 0.041 (0.998) | y = 0.0079x + 0.0048  (0.997) | -1905.49 | -10.21 |
| Propranolol | Pronethalol |  | y = 0.053x - 0.023  (1.000) | y = 0.053x + 0.012  (1.000) | 261.54 | 0.32 |
| Fluoxetine | Talopram |  | y = 0.012x - 0.0016  (1.000) | y = 0.012x + 0.0017  (1.000) | 106.33 | 3.20 |
| Acetaminophen | Acetaminophen-(*methyl*-d_3_) | 5-400 | y = 0.010x - 0.046  (0.997) | y = 0.0098x - 0.0051  (0.995) | 2527.36 | 931.43 |
| Carbamazepine | 10,11-Dihydrochloride | 1-400 | y = 0.0067x + 0.022 (0.999) | y = 0.0068x + 0.0077  (0.999) | -843.13 | 7.24 |
| BAC-C12 | BAC-C14-d_7_ | 2-80 | y = 0.0340x - 0.0806  (0.987) | y = 0.0318x + 0.0015  (0.984) | 329.68 | 0.0022 |
| BAC-C14 | BAC-C14-d_7_ |  | y = 0.0620x - 0.0824  (0.983) | y = 0.0598x - 0.0008  (0.989) | 218.69 | 6.07 |
| HDTMA | BAC-C14-d_7_ |  | y = 0.0552x - 0.0689  (0.969) | y = 0.0524x + 0.034  (0.977) | 302.77 | 0.0017 |

**Table S6:** Mass spectrometric and 1/x weighted quantitative data, linearity represented by the coefficient of determination, R^2^, limit of detection (LOD), mean percentage accuracy and precision of quality control (QC) samples for each analyte (acidic, neutral, basic n=3; amphiphilic n=5). Concentration of QCs are as follows, VLQC = very low QC, LQC = low QC, MQC = mid QC, UQC = upper QC.

| **Analyte** | **Regression Function** | **Linearity**  **(R^2^)** | **LOD (ng/mL)** | **Accuracy (%)** | | | | **Precision (%)** | | | |
| --- | --- | --- | --- | --- | --- | --- | --- | --- | --- | --- | --- |
|  |  |  |  | **VLQC** | **LQC** | **MQC** | **HQC** | **VLQC** | **LQC** | **MQC** | **HQC** |
| Diclofenac | 1/x | 0.998 | 1.039 | -2.9 | 4.4 | 3.4 | 3.4 | 8.6 | 10.5 | 4.4 | 0.8 |
| Loratadine | 1/x | 0.998 | 0.471 | 0.3 | -3.0 | -3.3 | -1.4 | 2.2 | 2.6 | 3.0 | 2.3 |
| Erythromycin | 1/x | 0.999 | 0.547 | 0.2 | 0.2 | -1.3 | -0.8 | 3.5 | 6.2 | 2.9 | 2.2 |
| Diphenhydramine | 1/x | 0.999 | 0.298 | 0.9 | 4.6 | 2.7 | 1.3 | 3.2 | 1.7 | 1.2 | 0.3 |
| Citalopram | 1/x | 0.997 | 0.220 | 12.2 | 16.0 | 9.6 | -1.4 | 2.6 | 2.8 | 2.1 | 0.9 |
| Propranolol | 1/x | 1.000 | 0.784 | 1.8 | 0.5 | -1.6 | 0.4 | 1.7 | 1.8 | 1.5 | 1.2 |
| Fluoxetine | 1/x | 1.000 | 0.142 | -2.4 | -1.1 | -0.7 | 1.6 | 3.3 | 4.5 | 4.5 | 1.1 |
| Acetaminophen | 1/x | 0.995 | 5.813 | -10.1 | -12.9 | -3.2 | 1.1 | 4.3 | 2.2 | 0.9 | 1.3 |
| Carbamazepine | 1/x | 0.999 | 0.508 | 7.3 | 7.1 | 3.9 | -1.9 | 1.8 | 2.5 | 1.6 | 1.0 |
| BAC-C12 | 1/x | 0.984 | 0.314 | -3.7 | -12.1 | -5.1 | -1.8 | 15.6 | 17.2 | 17.9 | 9.6 |
| BAC-C14 | 1/x | 0.989 | 0.278 | -2.1 | -1.3 | -0.6 | -2.9 | 11.2 | 16.9 | 20.5 | 13.5 |
| HDTMA | 1/x | 0.977 | 1.875 | 12.7 | -2.8 | -3.7 | -1.8 | 8.6 | 14.7 | 11.2 | 7.4 |

**Table S7:** Mean percentage matrix effects, recovery and process efficiency of each analyte and internal standards calculated using the spiked solvent samples following QuEChERS extraction (n=3).

| **Analytes** | | **Matrix effects, recovery and process efficiency of analytes in a spiked solvent extract** | | | | | | | | |
| --- | --- | --- | --- | --- | --- | --- | --- | --- | --- | --- |
|  |  | **Matrix effects** | | | | **Recovery** | | | | **Process efficiency** |
|  |  | **Mean (%)** | **Std Dev** | **%RSD** | **Std Error** | **Mean (%)** | **Std Dev** | **%RSD** | **Std Error** | **Mean (%)** |
| Acidic | Diclofenac | 109.2 | 0.06 | 5.79 | 0.04 | 2.6 | 0.01 | 57.40 | 0.01 | 2.8 |
|  | Loratadine | 102.1 | 0.10 | 9.66 | 0.06 | 41.4 | 0.02 | 5.68 | 0.01 | 42.1 |
| Neutral | Erythromycin | 109.2 | 0.16 | 14.24 | 0.09 | 32.9 | 0.05 | 15.36 | 0.03 | 35.5 |
|  | Diphenhydramine | 123.8 | 0.14 | 11.36 | 0.08 | 44.1 | 0.07 | 15.26 | 0.04 | 54.1 |
| Basic | Citalopram | 115.0 | 0.13 | 11.04 | 0.07 | 40.0 | 0.07 | 18.16 | 0.04 | 45.4 |
|  | Propranolol | 120.9 | 0.11 | 9.08 | 0.06 | 40.7 | 0.06 | 14.61 | 0.03 | 48.8 |
|  | Fluoxetine | 98.1 | 0.09 | 9.26 | 0.05 | 41.0 | 0.04 | 10.44 | 0.02 | 40.0 |
|  | Acetaminophen | 90.6 | 0.06 | 6.35 | 0.03 | 61.5 | 0.07 | 11.19 | 0.04 | 55.5 |
|  | Carbamazepine | 106.7 | 0.15 | 13.83 | 0.09 | 43.4 | 0.06 | 14.67 | 0.04 | 45.6 |
| Amphiphilic | BAC-C12 | 106.6 | 0.11 | 10.75 | 0.07 | 56.4 | 0.07 | 12.88 | 0.04 | 60.6 |
|  | BAC-C14 | 102.6 | 0.07 | 7.23 | 0.04 | 54.9 | 0.07 | 12.39 | 0.04 | 56.3 |
|  | HDTMA | 124.5 | 0.16 | 12.92 | 0.09 | 59.5 | 0.06 | 10.54 | 0.04 | 73.8 |
| Internal standards | Acet-(*methyl*-d_3_) | 164.9 | 0.23 | 13.94 | 0.13 | 62.9 | 0.04 | 5.71 | 0.02 | 103.2 |
|  | Pronethalol | 97.4 | 0.07 | 6.84 | 0.04 | 44.6 | 0.07 | 14.95 | 0.04 | 43.1 |
|  | 10,11-DHC | 102.2 | 0.09 | 9.07 | 0.05 | 45.9 | 0.03 | 7.60 | 0.02 | 46.7 |
|  | Talopram | 143.0 | 0.20 | 14.18 | 0.12 | 39.8 | 0.08 | 18.99 | 0.04 | 56.0 |
|  | BAC-C14-d_7_ | 94.6 | 0.05 | 4.87 | 0.03 | 56.8 | 0.10 | 16.94 | 0.06 | 53.8 |

**Table S8:** Mean percentage matrix effects, recovery and process efficiency of each analyte and internal standards calculated using the spiked soil samples following QuEChERS extraction (n=3).

| **Analytes** | | **Matrix effects, recovery and process efficiency of analytes in a spiked soil** | | | | | | | | |
| --- | --- | --- | --- | --- | --- | --- | --- | --- | --- | --- |
|  |  | **Matrix effects** | | | | **Recovery** | | | | **Process efficiency** |
|  |  | **Mean (%)** | **Std Dev** | **%RSD** | **Std Error** | **Mean (%)** | **Std Dev** | **%RSD** | **Std Error** | **Mean (%)** |
| Acidic | Loratadine | 99.5 | 0.10 | 9.80 | 0.06 | 64.4 | 0.03 | 4.87 | 0.02 | 64.0 |
| Neutral | Erythromycin | 122.6 | 0.28 | 22.84 | 0.16 | 48.4 | 0.05 | 11.26 | 0.03 | 58.3 |
|  | Diphenhydramine | 133.3 | 0.12 | 8.82 | 0.07 | 39.8 | 0.04 | 10.72 | 0.02 | 52.9 |
| Basic | Citalopram | 136.2 | 0.14 | 10.03 | 0.08 | 52.5 | 0.04 | 6.69 | 0.02 | 71.3 |
|  | Propranolol | 124.8 | 0.12 | 9.69 | 0.07 | 42.1 | 0.03 | 7.10 | 0.02 | 52.6 |
|  | Fluoxetine | 91.5 | 0.07 | 7.32 | 0.04 | 48.1 | 0.04 | 8.61 | 0.02 | 44.1 |
|  | Acetaminophen | 110.4 | 0.10 | 8.82 | 0.06 | 72.1 | 0.12 | 17.22 | 0.07 | 80.2 |
|  | Carbamazepine | 97.7 | 0.05 | 4.97 | 0.03 | 68.1 | 0.03 | 4.58 | 0.02 | 66.5 |
| Amphiphilic | BAC-C12 | 113.7 | 0.13 | 11.47 | 0.08 | 53.5 | 0.09 | 17.48 | 0.05 | 60.1 |
|  | BAC-C14 | 109.3 | 0.16 | 14.67 | 0.09 | 62.3 | 0.11 | 17.76 | 0.06 | 67.0 |
|  | HDTMA | 111.9 | 0.10 | 9.18 | 0.06 | 46.8 | 0.07 | 15.20 | 0.04 | 52.0 |
| Internal standards | Acet-(*methyl*-d_3_) | 141.8 | 0.03 | 1.96 | 0.02 | 100.1 | 0.12 | 12.21 | 0.07 | 141.8 |
|  | Pronethalol | 97.3 | 0.09 | 8.87 | 0.05 | 45.6 | 0.05 | 11.44 | 0.03 | 44.5 |
|  | 10,11-DHC | 92.4 | 0.01 | 0.84 | 0.00 | 66.7 | 0.01 | 1.53 | 0.01 | 61.6 |
|  | Talopram | 140.2 | 0.05 | 3.35 | 0.03 | 56.1 | 0.03 | 6.01 | 0.02 | 78.7 |
|  | BAC-C14-d_7_ | 113.5 | 0.01 | 0.45 | 0.00 | 55.3 | 0.09 | 16.75 | 0.05 | 62.8 |

**Table S9:** Mean percentage matrix effects, recovery and process efficiency of each pharmaceutical and internal standards calculated using the micro-QuEChERS extraction of spiked soil samples (n=3).

| **Analytes** | | **Matrix effects, recovery and process efficiency of analytes in a spiked soil using micro-QuEChERS** | | | | | | | | |
| --- | --- | --- | --- | --- | --- | --- | --- | --- | --- | --- |
|  |  | **Matrix effects** | | | | **Recovery** | | | | **Process efficiency** |
|  |  | **Mean (%)** | **Std Dev** | **%RSD** | **Std Error** | **Mean (%)** | **Std Dev** | **%RSD** | **Std Error** | **Mean (%)** |
| Acidic | Loratadine | 95.4 | 0.066 | 6.92 | 0.04 | 48.7 | 0.032 | 6.62 | 0.02 | 46.4 |
| Neutral | Erythromycin | 81.6 | 0.582 | 71.32 | 0.34 |  |  |  |  |  |
|  | Diphenhydramine | 86.0 | 0.061 | 7.07 | 0.04 | 27.1 | 0.043 | 16.05 | 0.03 | 23.1 |
| Basic | Citalopram | 83.6 | 0.042 | 5.03 | 0.02 | 28.3 | 0.030 | 10.55 | 0.02 | 23.6 |
|  | Propranolol | 97.0 | 0.265 | 27.29 | 0.15 | 31.6 | 0.090 | 28.58 | 0.05 | 29.1 |
|  | Fluoxetine | 89.0 | 0.148 | 16.63 | 0.09 | 25.8 | 0.070 | 26.96 | 0.04 | 22.3 |
|  | Acetaminophen |  |  |  |  |  |  |  |  |  |
|  | Carbamazepine | 92.3 | 0.091 | 9.82 | 0.05 | 49.7 | 0.079 | 15.97 | 0.05 | 45.6 |
| Amphiphilic | BAC-C12 | 104.6 | 0.149 | 14.26 | 0.09 | 58.5 | 0.091 | 15.49 | 0.05 | 60.4 |
|  | BAC-C14 | 87.0 | 0.008 | 0.93 | 0.00 | 57.4 | 0.047 | 8.17 | 0.03 | 49.9 |
|  | HDTMA | 103.2 | 0.101 | 9.75 | 0.06 | 33.4 | 0.037 | 11.19 | 0.02 | 34.3 |
| Internal standards | Acet-(*methyl*-d_3_) |  |  |  |  |  |  |  |  |  |
|  | Pronethalol | 87.0 | 0.109 | 12.47 | 0.06 | 35.4 | 0.047 | 13.25 | 0.03 | 30.5 |
|  | 10,11-DHC | 81.4 | 0.121 | 14.85 | 0.07 | 57.0 | 0.102 | 17.96 | 0.06 | 45.6 |
|  | Talopram | 90.7 | 0.144 | 15.88 | 0.08 | 25.5 | 0.018 | 7.22 | 0.01 | 22.9 |
|  | BAC-C14-d_7_ | 99.8 | 0.127 | 12.75 | 0.07 | 55.1 | 0.121 | 21.95 | 0.07 | 54.5 |

**Table S10:** Comparison of percentage mean matrix effects for the analytes spiked in soil using QuEChERS and micro-QuEChERS (n=3).

| **Analytes** | | **Matrix effects, recovery and process efficiency of analytes in a spiked soil** | |
| --- | --- | --- | --- |
|  |  | **QuEChERS** | **Micro-QuEChERS** |
|  |  | **ME (%)** | **ME (%)** |
| Acidic | Loratadine | -0.5 | -4.6 |
| Neutral | Erythromycin | +22.6 | -18.4 |
|  | Diphenhydramine | +33.3 | -14.0 |
| Basic | Citalopram | +36.2 | -16.4 |
|  | Propranolol | +24.8 | -3.0 |
|  | Fluoxetine | -8.5 | -11.0 |
|  | Acetaminophen | +10.4 |  |
|  | Carbamazepine | -2.3 | -7.7 |
| Amphiphilic | BAC-C12 | +13.7 | +4.6 |
|  | BAC-C14 | +9.3 | -13.0 |
|  | HDTMA | +11.9 | +3.2 |
| Internal standards | Acet-(*methyl*-d_3_) | +41.8 |  |
|  | Pronethalol | -2.7 | -13.0 |
|  | 10,11-DHC | -7.6 | -18.6 |
|  | Talopram | +40.2 | -9.3 |
|  | BAC-C14-d_7_ | +13.5 | -0.2 |

**Table S10:** Mean percentage matrix effects, recovery and process efficiency of each pharmaceutical and internal standards calculated using the micro-QuEChERS extraction for spiked plasma samples (n=3).

| **Analytes** | | **Matrix effects, recovery and process efficiency of analytes in a spiked plasma** | | | | | | | | |
| --- | --- | --- | --- | --- | --- | --- | --- | --- | --- | --- |
|  |  | **Matrix effects** | | | | **Recovery** | | | | **Process efficiency** |
|  |  | **Mean (%)** | **Std Dev** | **%RSD** | **Std Error** | **Mean (%)** | **Std Dev** | **%RSD** | **Std Error** | **Mean (%)** |
| Acidic | Loratadine | 88.2 | 0.130 | 14.76 | 0.08 | 83.1 | 0.057 | 6.85 | 0.03 | 72.8 |
| Neutral | Erythromycin |  |  |  |  |  |  |  |  |  |
|  | Diphenhydramine | 89.5 | 0.167 | 18.67 | 0.10 | 78.7 | 0.103 | 13.09 | 0.06 | 69.3 |
| Basic | Citalopram | 81.0 | 0.140 | 17.29 | 0.08 | 85.8 | 0.128 | 14.92 | 0.07 | 68.5 |
|  | Propranolol | 95.4 | 0.152 | 15.92 | 0.09 | 74.0 | 0.126 | 17.03 | 0.07 | 70.4 |
|  | Fluoxetine | 74.5 | 0.050 | 6.69 | 0.03 | 85.6 | 0.044 | 5.17 | 0.03 | 63.8 |
|  | Acetaminophen |  |  |  |  |  |  |  |  |  |
|  | Carbamazepine | 95.8 | 0.199 | 20.73 | 0.11 | 80.1 | 0.157 | 19.55 | 0.09 | 74.9 |
| Amphiphilic | BAC-C12 | 97.9 | 0.204 | 20.86 | 0.12 | 71.5 | 0.101 | 14.10 | 0.06 | 68.7 |
|  | BAC-C14 | 83.6 | 0.092 | 11.03 | 0.05 | 71.2 | 0.042 | 5.84 | 0.02 | 59.3 |
|  | HDTMA | 96.1 | 0.079 | 8.19 | 0.05 | 69.0 | 0.042 | 6.03 | 0.02 | 66.1 |
| Internal standards | Acet-(*methyl*-d_3_) |  |  |  |  |  |  |  |  |  |
|  | Pronethalol | 95.5 | 0.106 | 11.10 | 0.06 | 65.4 | 0.130 | 19.93 | 0.08 | 61.5 |
|  | 10,11-DHC | 83.3 | 0.111 | 13.29 | 0.06 | 79.7 | 0.105 | 13.22 | 0.06 | 65.6 |
|  | Talopram | 87.5 | 0.113 | 12.88 | 0.07 | 74.8 | 0.086 | 11.46 | 0.05 | 64.8 |
|  | BAC-C14-d_7_ | 93.4 | 0.157 | 16.81 | 0.09 | 75.6 | 0.080 | 10.56 | 0.05 | 70.1 |
